# Managing risks in drug discovery: reproducibility of published findings

**DOI:** 10.1007/s00210-016-1216-8

**Published:** 2016-02-17

**Authors:** Aimo Kannt, Thomas Wieland

**Affiliations:** Sanofi Diabetes Research and Development, Frankfurt, Germany; Institute of Experimental and Clinical Pharmacology and Toxicology, Medical Faculty Mannheim, Heidelberg University, Mannheim, Germany

**Keywords:** Risk management, Research and development, Reproducibility, Technical risk, Translational risk

## Abstract

In spite of tremendous advances in biopharmaceutical science and technology, the productivity of pharmaceutical research and development has been steadily declining over the last decades. The reasons for this decline are manifold and range from improved standard of care that is more and more difficult to top to inappropriate management of technical and translational risks along the R&D value chain. In this short review, major types of risks in biopharmaceutical R&D and means to address them will be described. A special focus will be on a risk, i.e., the lack of reproducibility of published information, that has so far not been fully appreciated and systematically analyzed. Measures to improve reproducibility and trust in published information will be discussed.

## The decline in pharmaceutical R&D productivity

The last decades have seen major advances and productivity gains in science and technology. The polymerase chain reaction (Saiki et al. 1985), for example, has revolutionized molecular biology and made it possible to amplify and quantify nucleic acids in a short time and at high throughput. Next-generation sequencing technologies (Ozsolak 2012) have reduced time and cost of whole-genome sequencing by several orders of magnitude from more than 10 years and three billion dollars for the first sequence of the human genome (Lander et al. 2001; Venter et al. 2001) to a few days and a thousand dollars (Hayden 2014), with further time and cost reductions being in sight. Computer power has increased exponentially: With well in excess of 10^11^ floating point operations per second (100 GFLOPS), modern smartphones are more than ten times more powerful than Deep Blue, the IBM supercomputer that beat chess world champion Garri Kasparow in 1997, that achieved 11.4 GFLOPS. The amount of sequencing, metabolomics, proteomics, microarray, and controlled-access human data available at the European Bioinformatics Institute approximately doubles every 12 months (Elixir 2014). RNA interference and gene-editing technologies have made it possible to investigate the role of individual genes and gene variants in complex systems in vitro and in vivo. The number and size of available chemical libraries have increased tremendously (Dolle 2011), and these compound libraries can be tested against protein targets at significantly higher throughput and lower costs (Mayr and Fuerst 2008).

**Fig. 1:**
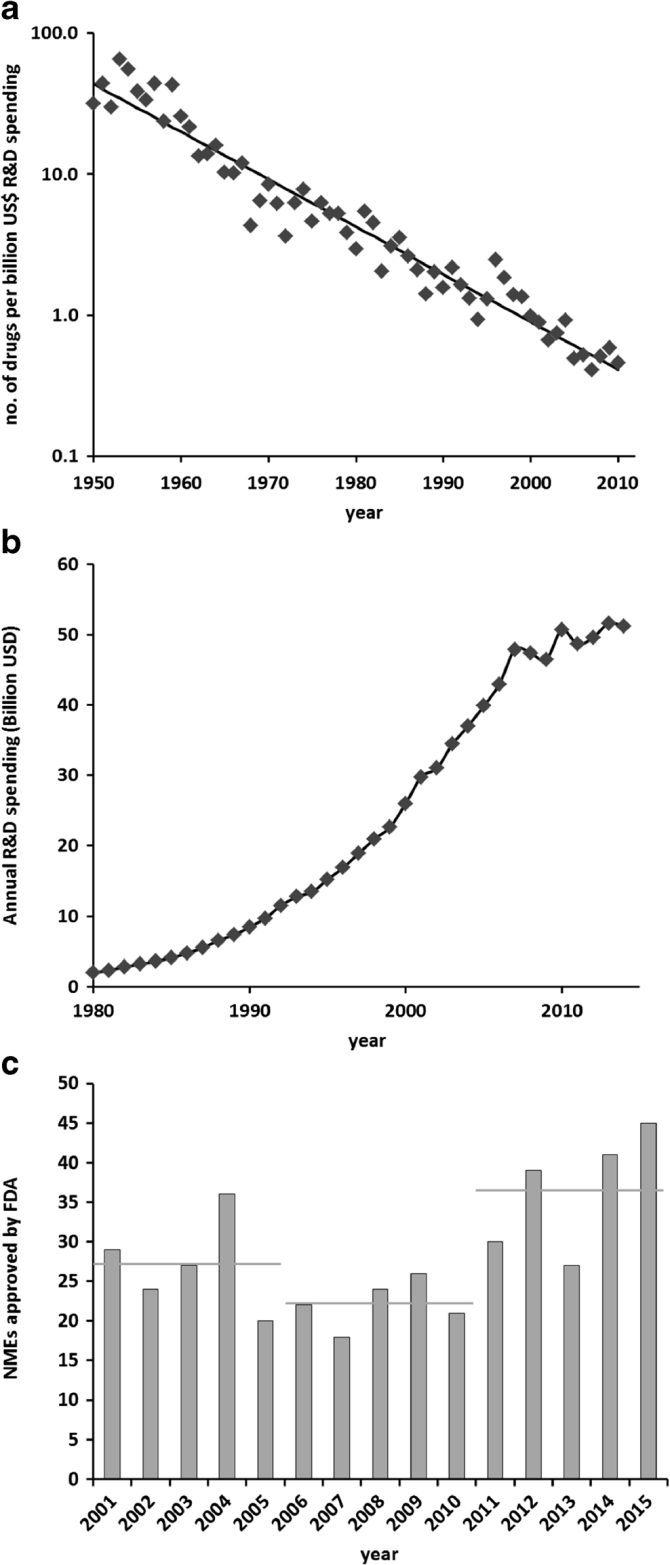
**a** “Eroom’s law” of declining R&D productivity in the pharmaceutical industry. The *straight line* denotes a decrease by a factor of 2 every 9 years. Data taken from Scannell et al. (2012), supplementary information. **b** Annual biopharmaceutical R&D spending by PhRMA member companies. Data from Phrma.org (2015). **c** Number of new molecular entities (NMEs) approved by the FDA for the years between 2001 and 2015. *Horizontal gray bars* denote the mean for the respective 5-year periods. Data from Phrma.org (2015) and fda.gov

Yet over the same period of time, pharmaceutical R&D has suffered from a steady decline in productivity. Whereas in other industries, output per invested amount of money has steadily improved, drug discovery and development have increasingly become more expensive, i.e., the amount of money to be invested for a new drug to be approved has approximately doubled every 9 years. This trend has been remarkably stable over the last six decades (see Fig. 1a). In analogy to “Moore’s law” that describes the exponential increase in productivity in the semiconductor industry based on the observation that the number of transistors on an integrated circuit approximately doubles every 2 years, this trend has been called “Eroom’s law”, Moore’s law in reverse (Scannell et al. 2012). Using different metrics, the numbers are even more disconcerting: setting the amount of research and development money spent in relation to the number of new drugs approved in the period between 1997 and 2011, it was estimated that true R&D costs per newly approved drug ranged from 3.7 to 11.8 billion US dollars in 12 major pharmaceutical companies (Herper 2012).

There are several potential reasons for this decline in R&D productivity: First, in many therapeutic fields, the standard of care is comparably efficacious and safe, setting a high bar for approval and re-imbursement of new drugs. This has been coined the “better than the Beatles” problem (Scannell et al. 2012) illustrating that every new drug has to be superior to all of what is already available. Second, health authorities have become more cautious of potential drug safety issues following, e.g., the cases of rofecoxcib and cerivastatin, and accordingly raised the bar for new treatments. For example, in 2008, the FDA issued guidance on the development of antidiabetics, requiring long-term cardiovascular safety trials to be performed for each new antidiabetic drug (FDA.gov 2008). Third, clinical trial failure rates have gone up considerably within a period of two decades (Mignani et al. 2015). Of note, the highest clinical attrition rates have been observed in phase II, with lack of efficacy being the major reason for failure (Hay et al. 2014; Cook et al. 2014). This is primarily due to insufficient target validation, lack of predictive preclinical models, or appropriate biomarkers. Failure rates were found to be particularly high in oncology with a likelihood of approval of only about 10 % for a phase 2 compound whereas it was nearly twice as high in the fields of endocrinology or infectious diseases (Hay et al. 2014). Key approaches to reduce later-stage clinical attrition are rigorous human target validation and early clinical proof-of-concept studies (Paul et al. 2010) to address these translational risks (see below). Fourth, the tendencies to streamline and industrialize pharmaceutical R&D thereby neglecting biological complexity have also contributed to clinical failures. Fifth, non-value-adding activities that are not on the critical path of a project lead to higher costs and longer timelines (Paul et al. 2010): Experiments or studies are done because they “can be done” or have traditionally been performed in previous approaches but have no impact on decision making within a specific project. Frequent changes in R&D strategy, re-organizations, and an inefficient bureaucracy with lengthy decision making processes also fall into this category of non-value-adding activities. Finally, and this is a major difference to other industries, cycle times in pharmaceutical R&D are, and will likely remain, very long: A project started today will not result in a product until 15 years—or often more—later. Within this time frame, projects may fail for technical or translational reasons (see below), but there are also many environmental changes like improvements in standard of care, new competitors, changes in regulatory requirements and medical care systems that may negatively influence the fate of a once promising idea or therapeutic concept and cannot always be foreseen when a project is initiated. Additionally, the outcome of pharmaceutical R&D is in most cases digital—a new drug product or no product. There is typically no equivalent to, e.g., a new instrument with a smaller footprint or a new car that consumes half a liter less per 100 km; it is a new drug or complete failure.

However, there is hope for better performance in future. Whereas the overall biopharmaceutical R&D spending seems to have reached a plateau (Phrma.org 2015; Fig. 1b), the number of new molecular entities (NMEs) approved by the FDA has increased in recent years, from an average of 22 per year between 2006 and 2010 to 36 between 2011 and 2015 (Fig. 1c). In a recent analysis, Smietana et al. (2015) used a novel metric, the so-called vintage index, to assess changes in R&D productivity over time. The index is defined as the revenue over 7 years of drugs launched in a given year divided by the R&D costs over the previous 7 years. Following a steady decline over the previous 15 years, the industry-wide vintage index nearly doubled between 2011 and 2014. These are encouraging signals that measures to recover R&D productivity have been successful, but longer-term data are needed to prove that this trend is sustainable and not just a transient deviation from Eroom’s law.

## Risk management in pharmaceutical R&D

Drug discovery is the management of risk. Embarking on a new drug discovery project can be the start of a journey that may last well over 12 years with a price tag in excess of several hundred million euros. Yet, more than 99 % of all drug discovery projects will not result in an approved product. Thus, most of the resources in pharmaceutical research and development will eventually not be spent on the few molecules making it to the market but primarily on the many molecules and projects that fail to do so. The reasons for failure can be manifold, from the wrong choice of target via the inability to find suitable lead compounds to unexpected toxicity or lack of efficacy in clinical trials. Moreover, projects may also be terminated for reasons that are not primarily based on science, like repeated strategy changes in pharmaceutical companies, changes in drug legislation and criteria or conditions for regulatory approval, a successful competitor, or weak intellectual property. And, even drugs that have been approved by the regulatory authorities may fail economically. Due to cost containment measures in the healthcare systems of many different countries, they simply will not give a sufficient return on the primary investment to foster further R&D.

Thus, when starting a new drug discovery project, potential risks have to be carefully evaluated and mitigation strategies to be developed. This applies to risks associated with the science aspects of the new project as well as the afore-mentioned strategic, regulatory, and commercial risks. For the part of science, two major types of risks can be distinguished: technical and translational risks.

As an attempt at a definition, technical risks can be described as those that lead to an inability to find and sufficiently characterize the right compound that meets the required profile to address the chosen target. Technical risks may present themselves as, for example, the lack of suitable bioassays, inability to generate the appropriate tools or reagents like cell models or antibodies, lack of selectivity toward the target, unsuitable pharmacokinetics, or toxicity of the generated molecules. A more comprehensive list of technical risks is given in Table 1.

**Table 1:** Selection of typical technical risks encountered in drug discovery

Type	Risks
Biochemistry, cell biology	No suitable assay for high-throughput screening or compound profiling
	Lack of cell permeability
Chemistry, biotechnology	No chemical or biological matter identified in HTS or other lead-finding approaches
	Lack of synthetic or biotechnological accessibility
	Insufficient solubility
	Limited potential for intellectual property
	High cost of goods
Pharmacology, pharmacokinetics	Insufficient potency on target
	Lack of exposure: limited bioavailability, rapid metabolization, unsuitable tissue distribution
	Limited in vivo efficacy
Safety	Potential for drug-drug interactions: CYP450 inhibition or induction
	Adverse effects in standard safety tests (e.g., hERG, genotoxicity)
	Toxicity

Translational risks, on the other hand, can be defined as those being responsible for insufficient clinical efficacy even for molecules having an otherwise perfect profile. This lack of efficacy can, for example, be the consequence of the wrong choice of target, the use of models that are not predictive for human disease, or failure of the molecule to engage the target in the clinical situation. Typical translational risks are summarized in Table 2.

**Table 2:** Selection of typical translational risks encountered in drug discovery and development

Translational risks	
• Target hypothesis based on false prerequisites; e.g., literature data that cannot be confirmed	
• Lack of cause-effect relationship: redundant pathways, counter-regulation upon knockout or inhibition	
• Limited understanding of disease biology and pathophysiological mechanisms	
• Preclinical models not predictive for clinical situation	
• Insufficient human evidence, e.g., from genetics or tool compounds	
• Lack of (surrogate) biomarkers to demonstrate target engagement and treatment efficacy	
• Inability to identify the right patient population for treatment	
• No add-on to existing therapy	

The most important difference between technical and translational risks is that the former can usually be mitigated by investing more resources, e.g., synthetizing and testing more molecules and repeated and different attempts at generating tools and models. Translational risks, however, cannot be addressed by more “shots on goal” but only by a rigorous and comprehensive investigation of the science behind the target, its link to human disease, identification of biomarkers to find the right patients amenable to the envisaged therapy, to monitor target engagement and to work as surrogates for clinical outcomes.

Notably, in a recent analysis of factors leading to failures in preclinical and clinical development (Cook et al. 2014), lack of clinical efficacy in phase II was determined as the most frequent cause of failure and insufficient target linkage to disease was identified as the most important reason for lack of clinical efficacy. Moreover, phase II success rate was nearly twofold higher with human genetics evidence linking the target to the disease indication and more than threefold higher when efficacy biomarkers were available at start of phase II. Thus, addressing translational risks very early, already at the target identification stage, will dramatically improve success rate in clinical development.

This insight has led to a paradigm shift in pharmaceutical drug discovery, away from a high-throughput, multiple shots on goal approach based on single target genes being regulated in animal models of disease to a more translational approach, starting with the interrogation of human disease biology and the identification of clinical biomarkers and then back-translating these findings into appropriate experimental model systems and molecules addressing these systems. Additionally, as a key approach to reduce phase II attrition, clinical proof of concept has to be established as early as possible, preferably in a phase I setting or even in parallel to preclinical drug discovery by, e.g., using approved molecules targeting the same mechanism and/or the use of biomarkers as surrogate endpoints (Paul et al. 2010). Thus, priority should be given to those targets with strong clinical evidence, appropriate reagents, and tools to model the clinical situation in a preclinical setting and the availability of biomarkers for target engagement, disease progression, or as surrogates for endpoints. To aid in prioritization, a translational scoring algorithm has been developed to systematically assess and quantify translational risks (Wehling 2009; Wendler and Wehling 2012).

A prominent example where selecting a drug target based on human genetics and available biomarkers has led to a rapid and successful generation and development of a drug is that of the secreted circulating protease proprotein convertase subtilisin/kexin type 9 (Pcsk9). In 2003, Pcsk9 mutations were identified in humans that cause familial hypercholesterolemia (Abifadel et al. 2003) via a gain-of-function mechanism resulting in overproduction of apoB100 (Ouguerram et al. 2004). Conversely, humans carrying a loss-of-function mutation in their Pcsk9 gene were demonstrated to have very low LDL levels and to be protected against coronary heart disease (Cohen et al. 2006). These findings prompted several companies to develop neutralizing monoclonal antibodies against Pcsk9 that have meanwhile demonstrated strong clinical efficacy in reducing LDL cholesterol and cardiovascular events on top of statin therapy (Robinson et al. 2015; Sabatine et al. 2015) and have received regulatory approval in the third quarter of 2015. Thus, two key points have led to new drugs being approved within less than 12 years after the publication of the first basic science findings: (1) strong clinical evidence, a so-called “experiment of nature,” demonstrating that loss of function of a particular gene provides clinical benefit without obvious adverse effects, and (2) biomarkers like LDL-C that allow for a rapid proof of concept and are accepted surrogate outcome markers.

## Reproducibility of published findings

Another important risk factor in biopharmaceutical R&D that has so far not been fully and systematically analyzed is the solidity and reproducibility of published data or, more precisely, the lack thereof. Typically, an interest in exploring a novel therapeutic target is sparked by literature data describing a role for this target in a disease context, predominantly in an accepted rodent model of disease. However, recent analyses by pharmaceutical industry researchers showed that the majority of these published studies could not be reproduced under well-controlled and standardized conditions. Prinz et al. (2011) investigated 57 drug discovery projects in the fields of cardiovascular disease, women’s health, or oncology and concluded that in more than two thirds of them there were major discrepancies to published data leading to project termination. Another analysis (Begley and Ellis 2012) came to even more dramatic conclusions: out of 53 landmark studies in the field of oncology under consideration, many of them published in high-profile journals, the primary scientific findings could only be reproduced in six (11 %) cases. In other studies, irreproducibility rates of 50 % and above have been found in a broader range of the biomedical literature (Vasilevsky et al. 2013; Hartsthorne and Schachner 2012). Although these estimates have to be taken with a grain of salt because the term “reproducibility” is not well defined, the enormous gap between these numbers and the conventionally assumed upper false-positive rate of 5 % (corresponding to *p* = 0.05) is alarming. Beside its scientific implications and the resulting lack of confidence and trust, it has a substantial economic impact: Freedman et al. recently estimated that, in the USA alone, 28 billion dollars are annually spent on preclinical research that is not reproducible (Freedman et al. 2015).

There are several potential reasons for this lack of reproducibility: First, no two experiments are the same. Especially living organisms are complex systems with a lot of variables like strain, age, sex, breeding and housing conditions, diet, et cetera, leading to variable results for studies being done by different researchers at different institutions, even when the overall study design and investigated parameters are very much comparable.

Second, improper study design and incorrect or inappropriate statistical analysis based on insufficient sample size may lead to non-reliable or even false conclusions (Prinz et al. 2011; Ioannidis 2005).

Third, biological reagents are frequently not sufficiently quality-controlled or inappropriately applied. Cell lines used in biomedical research, for example, are often misidentified or contaminated (Lorsch et al. 2014). A study on 122 different cancer cell lines showed that 30 % of them were not correctly identified (Zhao et al. 2011). Multidrug-resistant MCF-7 breast adenocarcinoma cells were used over two decades and in over 300 studies before they were demonstrated to be ovarian adenocarcinoma cells (Freedman et al. 2015; Liscovitch and Ravid 2007).

Fourth, research is hypothesis-driven, and researchers are often inclined to give findings supporting their hypothesis a stronger weight compared with experimental results that are in contradiction to their hypothesis. It is a widespread phenomenon that negative outcomes are only rarely published (Fanelli 2012; Kyzas et al. 2007; Sena et al. 2010). This hypothesis bias is reinforced by the policy of most scientific publishers and journals only to publish results that come with a good story line and reject findings or datasets leading to an ambiguous outcome.

Fifth, there is enormous competition and pressure from supervisors and financing bodies to present complete and conclusive data, and continuation of employment or funding of future work often depends on publications in high-impact journals. It has been reported that the likelihood of a paper supporting a tested hypothesis is higher when the corresponding author was working in a very competitive environment (Fanelli 2010) where publication pressure may have been higher. In the most extreme, this can lead to willful disregard of contradictory data or even downright data fabrication.

Therefore, the following practical measures should be taken to improve replicability and reproducibility of published findings. It is needless to say that they cannot be implemented and “lived” by a single party but require active participation by all stake holders involved, including but not limited to investigators, supervisors, reviewers, editors, funding bodies, and committees (Landis et al. 2012).

1. *Develop a “confirmation mindset”*: Before engaging in further studies, especially in a long and costly drug discovery project, it is mandatory to repeat published studies providing the project’s biological rationale and confirm their major conclusions. Ideally, this should be incentivized by journals and funding agencies. Instead of being a frequent reason for rejection of a manuscript, the first independent replication of an already published finding should be acceptable for publication, too. Alternatively, for a potential high-profile publication, journals may ask for confirmation of the major results by an independent party before accepting a manuscript for publication. Also, funding agencies or resource allocation committees ought to prioritize proposals seeking to confirm published data before performing extensive and costly follow-up experiments. Thus, future grants may provide a first tranche of financial support to replicate important former findings before granting the second, bigger tranche of money for continuing investigations based on already published data which did not obtain independent confirmation so far.
2. *Full transparency on protocols, materials and methods*: Ensure that all experimental details necessary to replicate a set of experiments are described comprehensively and that protocols, analysis plans, and raw data are included whenever possible. Most journals provide space in online supplements to include this information. Recommendations of standards for rigorous study design, conduct, and analysis have been published (Landis et al. 2012; Ioannidis et al. 2014). However, it is still not uncommon to find papers in which even very basic information is not provided, like strain, age, and gender of animals used in the study, composition of the diet, and whether and how animals were randomized or the study was blinded.
3. *Quality control*: Establish quality control procedures for reagents and materials used in experimental studies. Encourage the use of validated reagents. Ensure that quality control and reagent validation are required by publishers (Freedman et al. 2015).
4. *Scientific rigor*: As described above, publication pressure and hypothesis bias have led researchers to seek short-term success by “getting the story right” rather than to go for a rigorous and robust approach that will take longer and may produce less spectacular but more valid results—a strategy to “rather be first than to be right” (Macleod et al. 2014). For this to change, investigators, promotion committees and funding bodies have to initiate a cultural change. Instead of an impact factor centric reward system, institutions and committees should also judge researchers on the methodological rigor and quality of their research and the reproducibility of their findings (Macleod et al. 2014). Moreover, more credit should be given for teaching and mentoring (Begley and Ellis 2012) and the training of the next generation of scientists to rigorously design, conduct, analyze, and report their studies.
5. *Robust study design and appropriate use of statistics*: Especially preclinical studies are often done in an exploratory setting, without a predefined primary outcome, unblinded, and on a small number of animals. Such experiments have to be viewed and interpreted as hypothesis generating rather than hypothesis testing (Landis et al. 2012). For robust hypothesis testing, preclinical studies should be performed in settings that resemble randomized controlled clinical trials, with appropriate sample size, stringent protocols, and predefined measures of success. This may even be taken a step further to multicenter studies at several individual laboratories that, like clinical trials, are centrally coordinated with data being processed and analyzed under a single protocol, as recently described for the testing of an anti-CD94d antibody in an experimental model of stroke (Llovera et al. 2015; Tymianski 2015).
  Also, it is startling how many papers are still published although they contain basic statistical mistakes (Vaux 2012). Therefore, institutions have to ensure that students and researchers are being trained on proper study design and use of statistics. Reviewers and editors have to pay special attention to verify the appropriate use of statistics. Especially high-impact journals are therefore to be encouraged to, before accepting manuscripts for publication, verify the validity of the statistical analyses by professional biomathematicians getting access to the experimental raw data. If appropriate, raw data should be provided in a supplement.
6. *Embrace ambiguity*: It is often the case that the results within a set of experiments testing a specific hypothesis are not fully consistent. However, in an environment where journals, promotion committees, and funding agencies strive for the perfect story and are not prepared to accept ambiguity, it is tempting to de-prioritize datasets that are not in line with the story and only to publish the parts of the work that support the working hypothesis and are consistent with each other. This not only will give a distorted picture but also may lead to misinterpretation and false conclusions for parts of the study. What is wrong with, for example, saying that “we have found a robust and very interesting phenotype, but the experiments interrogating its molecular mechanism have led to contradictory findings and not given a clear result”? Such a statement would make clear what is robust and what deserves further investigation, dialogue with other researchers, and perhaps collaborative efforts to solve. Investigators, journals, and funding bodies should realize that nothing is wrong with such a statement, that it often reflects reality, and that gaps in stories may provide opportunities for further research (Begley and Ellis 2012).

## Conclusions

Drug discovery has changed significantly over the last decade. Previously, drug discovery was a process-driven and technology-oriented, almost factory-like high-throughput approach starting with a gene regulated in an animal model of disease (or the parallel interrogation of many disease-related genes) via the identification and optimization of compounds to their characterization in preclinical models and finally in the clinics, where, however, phase 2 attrition rates have been very high (Scannell et al. 2012; Cook et al. 2014). To reduce attrition at the clinical proof-of-concept stage, this “multiple shots on goal” paradigm is gradually being replaced by a more translational approach, where solid clinical evidence of target relevance, the availability of biomarkers, and a thorough mechanistic understanding of the link between target and disease are sought after very early in the drug discovery process and where projects are ranked according to translatability rather than technical feasibility or “druggability.”

Besides these process alterations, there are also cultural and mindset changes that will help to further reduce the cost of drug discovery research and hence improve its productivity. In apparent contrast to widespread scientific practice, experiments should be designed and executed to clearly demonstrate that the initial hypothesis is *wrong*—so called No-Go experiments—as failing early will save time and resources that would otherwise have been spent in vain. Such a “fail early” paradigm should also be reflected in the company culture and incentives for researches to stop projects based on rigorous science and to mark this as success rather than failure.

Target selection—the most important decision in a drug discovery project—is often based on literature data. However, various analyses have shown that a majority of published findings could not be reproduced by others. Therefore, replication of crucial studies is mandatory before embarking on a long and costly drug discovery program. Furthermore, all stakeholders involved in biomedical research should undertake efforts to recognize and reward reproducibility, to accept and even embrace ambiguity as this will not only improve confidence and trust but also stimulate further research and scientific dialogue.

## References

[CR1] Abifadel M, Varret M, Rabes JP, Allard D, Ouguerram K, Devillers M (2003). Mutations in Pcsk9 cause autosomal dominant hypercholesterolemia. Nat Genet.

[CR2] Begley CG, Ellis LM (2012). Drug development: raise standards for preclinical cancer research. Nature.

[CR3] Cohen JC, Boerwinkle E, Mosley TH, Hobbs HH (2006). Sequence variations in Pcsk9, low LDL, and protection against coronary heart disease. NEJM.

[CR4] Cook D, Brown D, Alexander R, March R, Morgan P, Satterthwaite G, Pangalos MN (2014). Lessons learned from the fate of AstraZeneca’s drug pipeline: a five-dimensional framework. Nat Rev Drug Discov.

[CR5] Dolle RE (2011). Historical overview of chemical library design. Methods Mol Biol.

[CR6] Elixir (2014) The ELIXIR Scientific Program 2014–2018. https://www.elixir-europe.org/system/files/elixir_scientific_programme_1.pdf accessed 13 Dec 2015

[CR7] Fanelli D (2010). Do pressures to publish increase scientists’ bias? An empirical support from US States Data. PLoS One.

[CR8] Fanelli D (2012). Negative results are disappearing from most disciplines and countries. Scientometrics.

[CR9] FDA.gov (2008) Guidance for industry: diabetes mellitus—evaluating cardiovascular risk in new antidiabetic therapies to treat type 2 diabetes http://www.fda.gov/downloads/Drugs/GuidanceComplianceRegulatoryInformation/Guidances/ucm071627.pdf accessed 15 October 2015

[CR10] Freedman LP, Cockburn I, Simcoe TS (2015). The economics of reproducibility in preclinical research. PLoS Biol.

[CR11] Hartsthorne JK, Schachner A (2012). Tracking replicability as a method of post-publication open evaluation. Front Comput Neurosci.

[CR12] Hay M, Thomas DW, Craighead JL, Economides C, Rosenthal J (2014). Clinical development success rates for investigational drugs. Nat Biotechnol.

[CR13] Hayden EC (2014). The 1000$ genome. Nature.

[CR14] Herper M (2012). The truly staggering costs of inventing new drugs.

[CR15] Ioannidis JP (2005). Why most published research findings are false. PLoS Med.

[CR16] Ioannidis JP, Greenland S, Hlatky MA, Khoury MJ, Macleod MR, Moher D (2014). Increasing value and reducing waste in research design, conduct, and analysis. Lancet.

[CR17] Kyzas PA, Denaxa-Kyza D, Ioannidis JPA (2007). Almost all articles on cancer prognostic markers report statistically significant results. Eur J Cancer.

[CR18] Lander ES, Linton LM, Birren B, Nusbaum C, Zody MC, Baldwin J (2001). The International Human Genome Sequencing Consortium. Initial sequencing and analysis of the human genome. Nature.

[CR19] Landis SC, Amara SG, Asadullah K, Austin CP, Blumenstein R, Bradley EW (2012). A call for transparent reporting to optimize the predictive value of preclinical research. Nature.

[CR20] Llovera G, Hofmann K, Roth S, Salas-Perdomo A, Ferrer-Ferrer M, Perego C (2015). Results of a preclinical randomized controlled multicenter trial (pRCT): anti-CD49d treatment for acute brain ischemia. Sci Transl Med.

[CR21] Lorsch JR, Collins FS, Lippincott-Schwartz J (2014). Cell biology. Fixing problems with cell lines. Science.

[CR22] Liscovitch M, Ravid D (2007). A case study in misidentification of cancer cell lines: MCF-7/AdrR cells (re-designated NCI/ADR-RES) are derived from OVCAR-8 human ovarian carcinoma cells. Cancer Lett.

[CR23] Macleod MR, Michie S, Roberts I, Dirnagl U, Chalmers I, Ioannidis JPA (2014). Biomedical research: increasing value, reducing waste. Lancet.

[CR24] Mayr LM, Fuerst P (2008). The future of high-throughput screening. J Biomol Scr.

[CR25] Mignani S, Huber S, Tomas H, Rodrigues J, Majoral JP (2015) Why and how have drug discovery strategies in pharma changed? What are the new mindsets? Drug Discovery Today. http://dx.doi.org/10.1016/j.drudis.2015.09.007 10.1016/j.drudis.2015.09.007 26376356

[CR26] Ouguerram K, Chetiveaux M, Zair Y, Costet P, Abifadel M, Varret M (2004). Apolipoprotein B100 metabolism in autosomal-dominant hypercholesterolemia related to mutations in PCSK9. Arterioscler Thromb Vasc Biol.

[CR27] Ozsolak F (2012). Third-generation sequencing techniques and applications to drug discovery. Expert Opin Drug Disc.

[CR28] Paul SM, Mytelka DS, Dunwiddie CT, Persinger CC, Munos BH, Lindborg SR, Schacht AL (2010). How to improve R&D productivity: the pharmaceutical industry’s grand challenge. Nat Rev Drug Discov.

[CR29] Phrma.org (2015) Biopharmaceutical research industry profile 2015. http://www.phrma.org/sites/default/files/pdf/2015_phrma_profile.pdf, accessed 28-10-2015

[CR30] Prinz F, Schlange T, Asadullah K (2011). Believe it or not: how much can we rely on published data on potential drug targets?. Nat Rev Drug Disc.

[CR31] Robinson JG, ODYSSEY LONG TERM investigators (2015). Efficacy and safety of alirocumab in reducing lipids and cardiovascular events. NEJM.

[CR32] Sabatine MS, Open-Label Study of Long-Term Evaluation against LDL Cholesterol (OSLER) Investigators (2015). Efficacy and safety of evolocumab in reducing lipids and cardiovascular events. NEJM.

[CR33] Saiki RK, Scharf S, Faloona F, Mullis KB, Horn GT, Erlich HA, Arnheim N (1985). Enzymatic amplification of beta-globin genomic sequences and restriction site analysis for diagnosis of sickle cell anemia. Science.

[CR34] Scannell JW, Blanckley A, Boldon H, Warrington B (2012). Diagnosing the decline in pharmaceutical R&D efficiency. Nature Rev Drug Disc.

[CR35] Sena ES, van der Worp HB, Bath PMW, Howels DW, Macleod MR (2010). Publication bias in reports of animal stroke studies leads to major overstatement of efficacy. PLoS Biol.

[CR36] Smietana K, Ekstrom L, Jefferey B, Moller M (2015). Improving R&D productivity. Nat Rev Drug Discov.

[CR37] Tymianski M (2015). Neuroprotective therapies: preclinical reproducibility is only part of the problem. Sci Transl Med.

[CR38] Vasilevsky NA, Brush MH, Paddock H, Ponting L, Tripathy SJ (2013). On the reproducibility of science: unique identification of research resources in the biomedical literature. Peer J.

[CR39] Vaux DL (2012). Know when your numbers are significant. Nature.

[CR40] Venter JC, Adams MD, Myers EW, Li PW, Mural RJ, Sutton GG (2001). The sequence of the human genome. Science.

[CR41] Wehling M (2009). Assessing the translatability of drug projects: what needs to be scored to predict success?. Nature Rev Drug Disc.

[CR42] Wendler A, Wehling M (2012). Translatability scoring in drug development: eight case studies. J Transl Med.

[CR43] Zhao M, Sano D, Pickering CR, Jasser SA, Henderson YC, Clayman GL (2011). Assembly and initial characterization of a panel of 85 genomically validated cell lines from diverse head and neck tumor sites. Clin Cancer Res.

